# Crystal structure of bis­(*N*,*N*′-di­methyl­thio­urea-κ*S*)bis­(thio­cyanato-κ*N*)cobalt(II)

**DOI:** 10.1107/S2056989020011111

**Published:** 2020-08-18

**Authors:** Aleksej Jochim, Rastko Radulovic, Inke Jess, Christian Näther

**Affiliations:** a Institut für Anorganische Chemie, Christian-Albrechts-Universität Kiel, Max-Eyth-Str. 2, D-24118 Kiel, Germany

**Keywords:** crystal structure, cobalt thio­cyanate, *N*,*N*′-di­methyl­thio­urea, discrete complexes, thermal properties

## Abstract

During systematic investigations on the synthesis of coordination polymers with Co(NCS)_2_ involving different thio­urea derivatives as coligands, crystals of the title compound Co(NCS)_2_(*N*,*N*′-di­methyl­thio­urea)_2_, or [Co(C_3_H_8_N_2_S)_2_(NCS)_2_], were obtained. These crystals were non-merohedric twins and therefore, a twin refinement using data in HKLF-5 format was performed. In the crystal structure of this compound, the Co^II^ cations are coordinated by two N-terminally bonded thio­cyanate anions as well as two S-bonding *N*,*N*′-di­methyl­thio­urea mol­ecules, forming two crystallographically independent discrete complexes each with a strongly distorted tetra­hedral geometry. An intricate network of inter­molecular N—H⋯S and C—H⋯S hydrogen bonds can be found between the complexes. The thermogravimetric curve of the title compound shows two discrete steps in which all coligand mol­ecules have been emitted, which is also accompanied by partial decomposition of the cobalt thio­cyanate. If the measurement is stopped after the first mass loss, only broad reflections of CoS can be found in the XRPD pattern of the residue, which proves that this compound decomposes completely upon heating. However, at lower temperatures an endothermic signal can be found in the DTA and DSC curve, which corresponds to melting, as proven by thermomicroscopy.

## Abstract 2

In the crystal structure of the title compound, Co(NCS)_2_(*N*,*N*′-di­methyl­thio­urea)_2_, discrete tetra­hedral complexes are found in which the thio­cyanate anions are N-terminally coordinated, while the coligands are S-terminally coordinated and are connected *via* weak hydrogen bonds into a three-dimensional structure.

## Chemical context   

Investigations on the synthesis, crystal structures and magnetic properties of coordination polymers based on transition-metal thio­cyanates have become of increasing inter­est in recent years, which is due to the high structural diversity of the thio­cyanate anion and its ability to mediate reasonable magnetic exchange. Although many different transition-metal thio­cyanate coordination compounds are known, our main inter­ests focus on first-row transition-metal thio­cyanate compounds with the general composition *M*(NCS)_2_(*L*)_2_ in which paramagnetic metal cations *M* are connected by pairs of μ-1,3 bridging thio­cyanate anions into chains, while the remaining coordination sites are occupied by neutral coligands *L*, forming an octa­hedral coordination polyhedron. In most of these compounds, linear chains are formed in which all ligands are *trans* (Prananto *et al.*, 2017 ▸; Werner *et al.*, 2015*a* ▸; Mautner *et al.*, 2018 ▸; Rams *et al.*, 2020 ▸; Jin *et al.*, 2007 ▸), but other isomers are also possible. This includes compounds in which either the N-bonding thio­cyanates, the S-bonding thio­cyanates or the coligands are *trans*, while the other ligands are *cis*. These motifs are found, for example, in [*M*(NCS)_2_(4-benzyl­pyridine)_2_]_*n*_ (*M* = Mn, Ni, Cd) (Suckert *et al.*, 2015 ▸; Jochim *et al.*, 2018 ▸; Neumann *et al.*, 2018*a* ▸), [*M*(NCS)_2_(4-nitro­pyridine *N*-oxide)_2_]_*n*_ (*M* = Mn, Co, Cd) (Shi *et al.*, 2006 ▸, 2007 ▸; Mautner *et al.*, 2016 ▸) and [*M*(NCS)_2_(4-benzoyl­pyridine)_2_]_*n*_ (*M* = Co, Ni) (Rams *et al.*, 2017 ▸; Jochim *et al.*, 2018 ▸), in which different *cis*–*cis*–*trans* configurations can be found around the metal centers. The last possible isomer, in which all ligands are *cis*, is not found for the composition *M*(NCS)_2_(*L*)_2_, although it was observed in more ligand-deficient compounds of composition Co(NCS)_2_(*L*), with *L* representing either 4-methyl­pyridine *N*-oxide or 4-meth­oxy­pyridine *N*-oxide (Zhang *et al.*, 2006*a* ▸,*b* ▸).

In those cases in which either only the N-bonding or the S-bonding thio­cyanate are *cis*, corrugated chains are formed instead of linear ones, which is also the case if all ligands are *cis*. Furthermore, in some compounds a mixture of these configurations can be found, as in [*M*(NCS)_2_(4-chloro­pyridine)_2_]_*n*_ (*M* = Co, Ni, Cd) (Böhme *et al.*, 2020 ▸; Jochim *et al.*, 2018 ▸; Goher *et al.*, 2003 ▸) or the high-temperature modification of [Ni(NCS)_2_(4-amino­pyridine)_2_]_*n*_ (Neumann *et al.*, 2018*b* ▸), in which an alternating arrangement of all-*trans* and *cis*–*cis*–*trans-*coordinated metal cations is present.

For some compounds of composition *M*(NCS)_2_(*L*)_2_ a completely different structure is observed, in which the metal cations are connected into layers of different topologies by the thio­cyanate anions. Only one topology is known for cobalt compounds of this composition, in which every two cobalt cations are connected by a pair of μ-1,3-bridging thio­cyanate anions, forming dimers, which are connected to four adjacent dimers by single μ-1,3-bridging thio­cyanate anions (Wöhlert & Näther, 2013 ▸; Suckert *et al.*, 2016 ▸, 2017 ▸; Werner *et al.*, 2015*b* ▸). Nevertheless, with other metal cations different layer topologies are known in which, for example, trimers are formed instead of dimers, which are connected by single μ-1,3-thio­cyanate bridges (Kozísková *et al.*, 1990 ▸; Kabešová *et al.*, 1990 ▸) or in which each metal cation is directly connected to four neighboring metal cations by single μ-1,3-thio­cyanate bridges (McElearney *et al.*, 1979 ▸; Werner *et al.*, 2015*c* ▸; Đaković *et al.*, 2010 ▸). Although it is not clear by which parameters the formation of either chains or layers is promoted, a third isomer of composition Co(NCS)_2_(*L*)_2_ exists in which the thio­cyanate anions are N-terminally coordinated, forming discrete tetra­hedral complexes (Prananto *et al.*, 2017 ▸; Neumann *et al.*, 2018*c* ▸; Hannachi *et al.*, 2019 ▸). In most of these compounds, electron-rich coligands are found, which might indicate that this type of compound is formed when a strong donor is used as coligand.

Most of the aforementioned compounds contain N-donor coligands, but in the course of our systematic work we became inter­ested in the influence of S-donor coligands based on thio­urea derivatives on the magnetic properties of Co(NCS)_2_ chain compounds, in which the metal cations are linked by pairs of μ-1,3-bridging thio­cyanate anions. With nickel, two compounds with the composition Ni(NCS)_2_(ethyl­ene­thio­urea)_2_ (Nardelli *et al.*, 1966 ▸) and Ni(NCS)_2_(thio­acetamide)_2_ (Capacchi *et al.*, 1968 ▸) are known. We prepared the corres­ponding cobalt compound with ethyl­ene­thio­urea, which is isotypic to the nickel analogue (Jochim *et al.*, 2020*a* ▸). Only one additional polymeric compound with the composition [Co(NCS)_2_(thio­urea)_2_]_*n*_ is known, in which the cobalt cations are connected by the sulfur atoms of the coligands, while the thio­cyanate anions are N-terminally coordinated (Rajarajan *et al.*, 2012 ▸; Muthu *et al.*, 2015 ▸). In further work we prepared Co(NCS)_2_(tetra­methyl­thio­urea)_2_, which surprisingly consists of discrete tetra­hedral complexes (Jochim *et al.*, 2020*b* ▸).
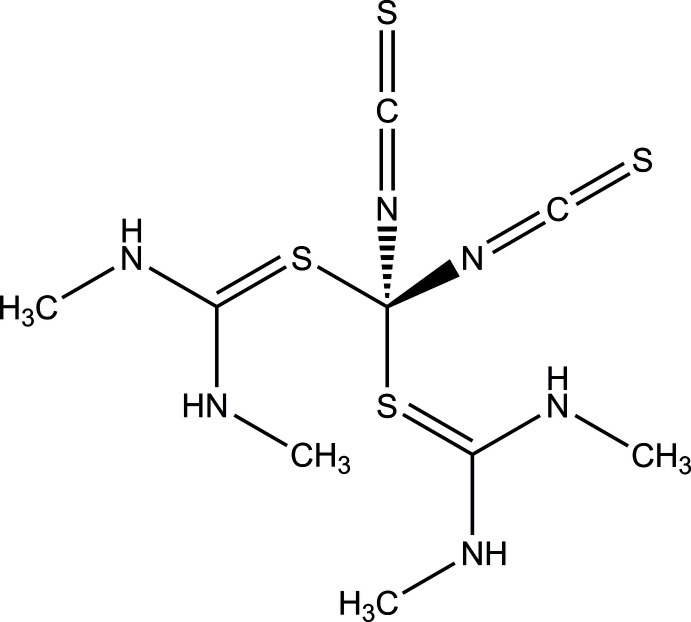


To further investigate this structural behavior, we used *N*,*N*′-di­methyl­thio­urea as coligand, which is between thio­urea and tetra­methyl­thio­urea considering the substitution with methyl groups, and during these investigations the title compound was obtained. Its IR spectrum shows that the C—N stretching vibration is found at 2064 cm^−1^, which is indicative of N-terminally bonded thio­cyanate anions, pointing to the formation of a tetra­hedral complex, as proven by single crystal X-ray diffraction (see Fig. S1 in the supporting information). Although the experimental XRPD pattern is very similar to that calculated for the title compound, some additional reflections are found, indicating some contamination (Fig. S2). This is surprising, because all samples were prepared in different ways and using a different ratio of Co(NCS)_2_ and di­methyl­thio­urea leads to an identical XRPD pattern. In the thermogravimetry curve (Fig. S3), two mass losses of 37.2% and 26.3% are observed, which in total is more than expected for the emission of both coligand mol­ecules (54.3%), indicating the decomposition of the thio­cyanate anions. This was proven by PXRD of the residue isolated after the first mass loss, which is mostly amorphous but which contains a small amount of crystalline CoS (Fig. S4). At low temperatures, an additional endothermic event can be found in the differential thermoanalysis curve, which is not accompanied by a mass change of the sample. This event corresponds to melting of the sample and is not reversible upon cooling, which was shown by differential scanning calorimetry (Fig. S5) and thermomicroscopy (Fig. S6).

## Structural commentary   

**Figure 1:**
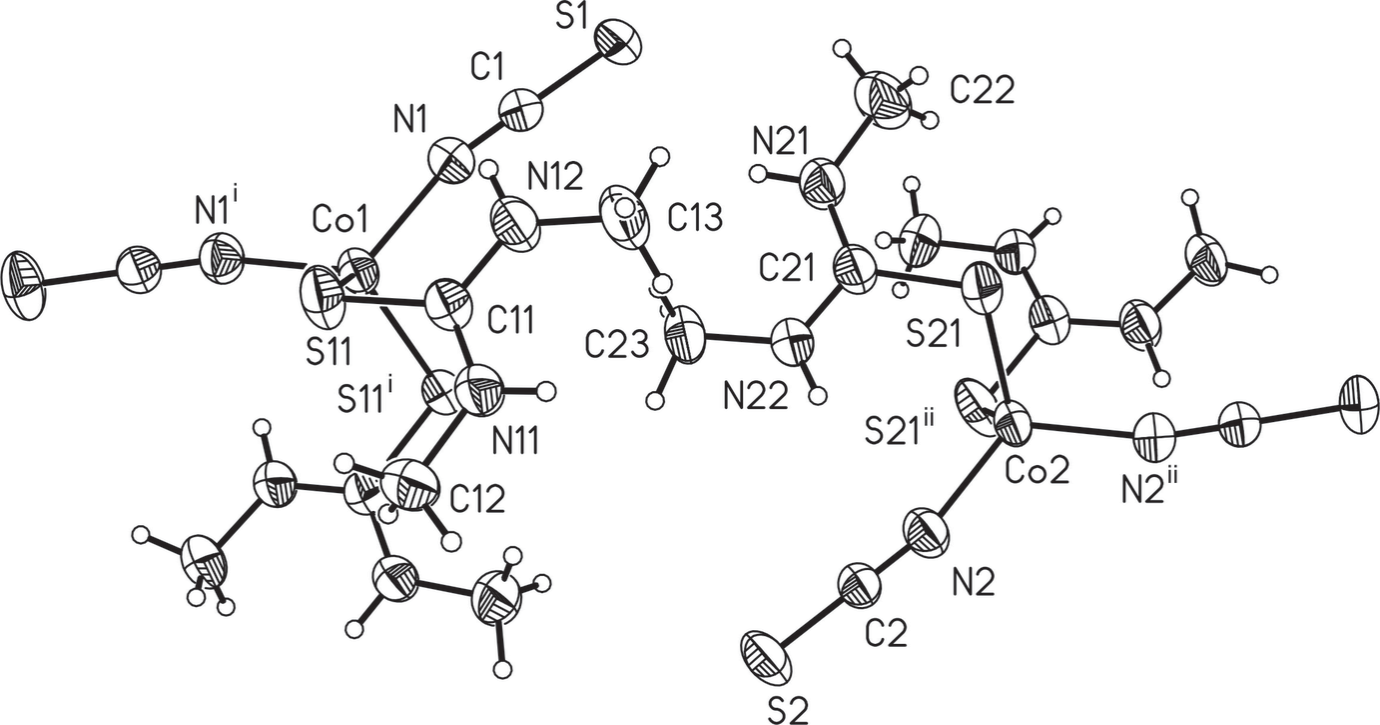
View of the two crystallographically independent molecules in the asymmetric unit of the title compound with atom labeling and displacement ellipsoids drawn at the 50% probability level. Symmetry codes: (i) −*x* + 1, *y*, −*z* + 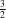; (ii) −*x*, *y*, −*z* + 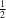.

The asymmetric unit of the title compound contains two crystallographically independent complexes built up of two thio­cyanate anions and two *N*,*N*′-di­methyl­thio­urea mol­ecules in general positions as well as one cobalt(II) cation, which is situated on a twofold rotational axis in both crystallo­graphically independent complexes. Both metal cations are coordinated by two N-bonding thio­cyanate anions and two S-bonding *N*,*N*′-di­methyl­thio­urea mol­ecules, forming strongly distorted tetra­hedra (Fig. 1 ▸), which becomes obvious from the tetra­hedral angle variance σ_θ〈tet〉_^2^ = 73.2 (Co1) or 73.3 (Co2) and the mean tetra­hedral quadratic elongation 〈λ_tet_〉 = 1.030 (Co1, Co2) (Robinson *et al.*, 1971 ▸). However, all bond lengths and angles are comparable to those reported for similar compounds in the literature (Neumann *et al.* 2018*c* ▸). Both *N*,*N*′-di­methyl­thio­urea mol­ecules are nearly planar with C_Me_—N—C—N torsion angles of 177.7 (3) and −0.5 (4)° for the complex containing Co1 and 178.9 (3) and −1.2 (4)° for that containing Co2. Both *N*,*N*′-di­methyl­thio­urea planes within each complex are slightly tilted relative to each other, leading to an angle of 9.51 (10)° between the normal vectors of the *N*,*N*′-di­methyl­thio­urea planes in the complex containing Co1, while the corresponding angle for the complex containing Co2 is 13.52 (8)°. Similar values are observed for the corresponding angles between the *N*,*N*′-di­methyl­thio­urea planes of different crystallographically independent complexes.

## Supra­molecular features   

**Figure 2:**
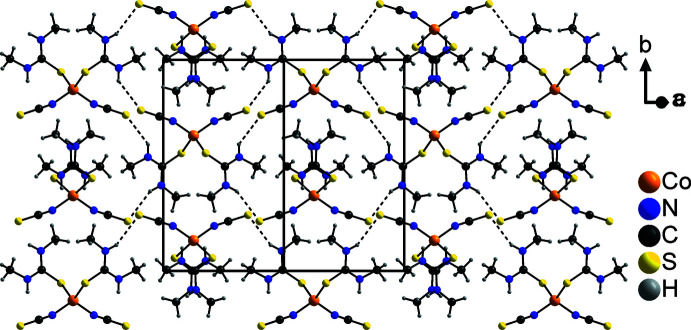
Crystal structure of the title compound with a view of the chains that extend along [101]. Inter­molecular N—H⋯S hydrogen bonding is shown as dashed lines.

**Figure 3:**
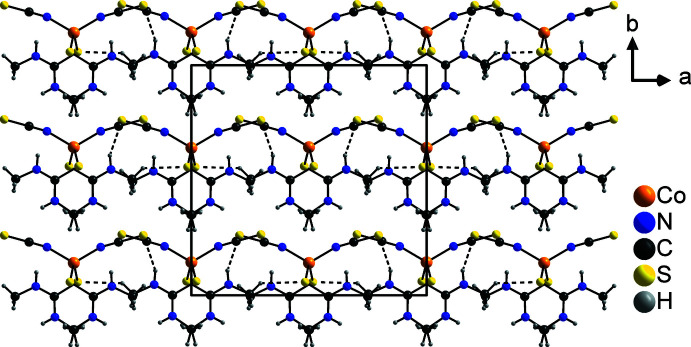
Crystal structure of the title compound with a view of the chains that extend along the *a*-axis direction. Inter­molecular N—H⋯S and C—H⋯S hydrogen bonds are shown as dashed lines.

**Figure 4:**
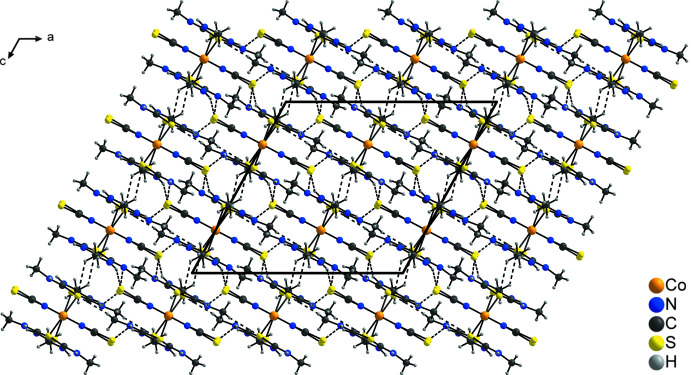
Crystal structure of the title compound with a view perpendicular to the layers parallel to the crystallographic *ac* plane. Inter­molecular N—H⋯S and C—H⋯S hydrogen bonds are shown as dashed lines.

The discrete complexes are connected *via* relatively weak N—H⋯S_NCS_ hydrogen bonds (Table 1 ▸), forming chains along the [101] direction in which the two crystallographically independent discrete complexes alternate. For both complexes, either only the thio­cyanate anions (Co1) or the *N*,*N*′-di­methyl­thio­urea mol­ecules (Co2) are involved in the formation of this structure. Two neighboring chains are connected into double chains by pairs of additional N—H⋯S_NCS_ hydrogen bonds involving only the complexes containing Co1 (Fig. 2 ▸). In a similar way, chains built up of an alternating sequence of complexes containing either Co1 or Co2 are formed, which run along the *a*-axis direction, but in this case, in the complexes containing Co1, only the *N*,*N*′-di­methyl­thio­urea mol­ecules participate in the N—H⋯S_NCS_ hydrogen bonds, while in the complex containing Co2, only the thio­cyanate anions are involved. Within these chains, additional C—H⋯S hydrogen bonds between the *N*,*N*′-di­methyl­thio­urea mol­ecules of adjacent complexes can be found (Fig. 3 ▸). The two types of chain are connected *via* C—H⋯S hydrogen bonds between the *N*,*N*′-di­methyl­thio­urea mol­ecules, forming layers parallel to the *ac* plane (Fig. 4 ▸), which are further connected into a three-dimensional network by additional N—H⋯S_NCS_ hydrogen bonds.

## Database survey   

In the Cambridge Structure Database (Version 5.41, last update November 2019; Groom *et al.*, 2016 ▸), only one trans­ition-metal thio­cyanate compound with *N*,*N*′-di­methyl­thio­urea is reported, which has the composition Cu(NCS)(*N*,*N*′-di­meth­yl­thio­urea)[propane-1,3-diylbis(di­phenyl­phosphine)] (Wattanakanjana *et al.*, 2015 ▸). In this compound, the Cu^I^ cations are tetra­hedrally coordinated by one thio­cyanate anion, one *N*,*N*′-di­methyl­thio­urea mol­ecule and one propane-1,3-diylbis(di­phenyl­phosphine) mol­ecule, with both phosphines coordinating to the metal center. In total, only 49 compounds containing a transition-metal cation and *N*,*N*′-di­methyl­thio­urea are known, none of which contains cobalt. In fact, 28 of these compounds involve more chalcophilic second or third row transition-metal cations, while nearly half of the remaining compounds contain copper.

Considering compounds that contain cobalt thio­cyanate, over one thousand compounds are found, most of which show either an octa­hedral or a tetra­hedral coordination geometry with the octa­hedral coordination geometry being the most common. Nevertheless, approximately 200 compounds with tetra­hedral coordination geometry are known, making it the second most common coordination geometry for cobalt thio­cyanate compounds.

## Synthesis and crystallization   

**General**

*N*,*N*′-di­methyl­thio­urea was purchased from Sigma Aldrich, while Co(NCS)_2_ was purchased from Alfa Aesar. All chemicals were used without further purification.

**Synthesis**

Single crystals were obtained by reacting Co(NCS)_2_ (0.15 mmol, 26.3 mg) with *N*,*N*′-di­methyl­thio­urea (0.3 mmol, 31.3 mg) in 0.4 mL of water. After approximately one week, deep-blue crystals were obtained, which were suitable for single crystal analysis. The same procedure was used to obtain powder samples using a higher amount of Co(NCS)_2_ (1.00 mmol, 175.1 mg), *N*,*N*′-di­methyl­thio­urea (2.0 mmol, 208.4 mg) and water (0.75 mL). The resulting crystals were ground into powder. In some cases, no crystallization of the mixture occurred, which was prevented by storing the reaction mixture at 281 K. Elemental analysis calculated for C_8_H_16_N_6_CoS_4_ (383.45 g/mol) C 25.06%, H 4.21%, N 21.92%, S 33.45%, found: C 24.79%, H 4.34%, N 21.75%, S 32.16%.

**Experimental details**

Elemental analysis was performed using a EURO EA elemental analyzer fabricated by EURO VECTOR Instruments.

The IR spectrum was measured using an ATI Mattson Genesis Series FTIR Spectrometer, control software: WINFIRST, from ATI Mattson.

The PXRD measurement was performed with Cu *K*α_1_ radiation (λ = 1.540598 Å) using a Stoe Transmission Powder Diffraction System (STADI P) equipped with a MYTHEN 1K detector and a Johansson-type Ge(111) monochromator.

DTA–TG measurements were performed in a dynamic nitro­gen atmosphere (100 sccm) in Al_2_O_3_ crucibles using a STA-PT 1600 thermobalance from Linseis. The instrument was calibrated using standard reference materials.

The DSC measurements were performed with a DSC 1 Star System with STARe Excellence Software from Mettler–Toledo AG. The instrument was calibrated using standard reference materials.

Thermomicroscopic measurements were performed using an FP82 hot stage from Mettler and a BX60 microscope from Olympus, using the analysis software package from Mettler.

## Refinement   

Crystal data, data collection and structure refinement details are summarized in Table 2 ▸. All non-hydrogen atoms were refined anisotropically. The C—H H atoms were positioned with idealized geometry and refined isotropically with *U*_iso_(H) = 1.5*U*_eq_(C), allowing them to rotate, but not to tip. The N—H H atoms were located in the difference map, their bond lengths were set to ideal values and finally they were refined using a riding model [*U*_iso_(H) = 1.2*U*_eq_(N)]. As the crystal studied was twinned by non-merohedry, a twin refinement using data in HKLF 5 format was performed. The corres­ponding files were generated by *PLATON* (Spek 2020 ▸).

## Supplementary Material

Crystal structure: contains datablock(s) I. DOI: 10.1107/S2056989020011111/tx2032sup1.cif

Structure factors: contains datablock(s) I. DOI: 10.1107/S2056989020011111/tx2032Isup2.hkl

Figure S1. IR spectrum of the title compound. Given is the value of the CN stretching vibration of the thiocyanate anions. DOI: 10.1107/S2056989020011111/tx2032sup3.tif

Figure S2. Experimental (top) and calculated (bottom) PXRD pattern of the title compound measured with Cu-radiation. DOI: 10.1107/S2056989020011111/tx2032sup4.tif

Figure S3. DTG, TG and DTA curve of the title compound measured with 4C/min. in a nitrogen atmosphere. DOI: 10.1107/S2056989020011111/tx2032sup5.tif

Figure S4. XRPD pattern of the residue obtained after the first mass loss (top) together with the calculated pattern for CoS. DOI: 10.1107/S2056989020011111/tx2032sup6.tif

Figure S5. DSC heating and cooling curve of the title compound until the first endothermic event using a heating rate of 4C/min. under a nitrogen atmosphere. DOI: 10.1107/S2056989020011111/tx2032sup7.tif

Figure S6. Thermomicroscopic images of the title compound at different temperatures when heated in air with 10 C/min. DOI: 10.1107/S2056989020011111/tx2032sup8.tif

CCDC reference: 2023019

Additional supporting information:  crystallographic information; 3D view; checkCIF report

## Figures and Tables

**Table 1:** Hydrogen-bond geometry (Å, °)

*D*—H⋯*A*	*D*—H	H⋯*A*	*D*⋯*A*	*D*—H⋯*A*
N11—H11⋯S1^i^	0.88	2.65	3.341 (2)	136
N12—H12⋯N1	0.88	2.69	3.282 (3)	126
N12—H12⋯S2^ii^	0.88	2.88	3.582 (2)	138
C13—H13*B*⋯S21^iii^	0.98	2.80	3.578 (3)	137
N21—H21⋯S1	0.88	2.61	3.372 (2)	146
C22—H22*A*⋯S2^iv^	0.98	3.01	3.591 (3)	120
C22—H22*A*⋯S21^iii^	0.98	3.02	3.752 (4)	132
N22—H22⋯S2^v^	0.88	2.79	3.486 (3)	137

Symmetry codes: (i) 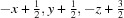; (ii) 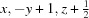; (iii) 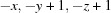; (iv) 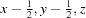; (v) 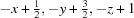.

**Table 2:** Experimental details

Crystal data	
Chemical formula	[Co(C_3_H_8_N_2_S)_2_(NCS)_2_]
*M*_r_	383.44
Crystal system, space group	Monoclinic, *C*2/*c*
Temperature (K)	200
*a*, *b*, *c* (Å)	16.8070 (8), 14.4212 (6), 15.5669 (9)
β (°)	118.744 (4)
*V* (Å^3^)	3308.1 (3)
*Z*	8
Radiation type	Mo *K*α
μ (mm^−1^)	1.54
Crystal size (mm)	0.10 × 0.08 × 0.07

Data collection	
Diffractometer	Stoe IPDS2
No. of measured, independent and observed [*I* > 2σ(*I*)] reflections	3218, 3218, 2767
(sin θ/λ)_max_ (Å^−1^)	0.618

Refinement	
*R*[*F* ^2^ > 2σ(*F* ^2^)], *wR*(*F* ^2^), *S*	0.039, 0.105, 1.07
No. of reflections	3218
No. of parameters	178
H-atom treatment	H-atom parameters constrained
Δρ_max_, Δρ_min_ (e Å^−3^)	0.72, −0.40

Computer programs: *X-AREA* and *XP* (Stoe & Cie, 2002 ▸), *SHELXS97* (Sheldrick, 2008 ▸), *SHELXL2018/3* (Sheldrick, 2015 ▸), *DIAMOND* (Brandenburg & Putz, 1999 ▸) and *publCIF* (Westrip, 2010 ▸).

## supplementary crystallographic information

### Crystal data

**Table:**

[Co(C_3_H_8_N_2_S)_2_(NCS)_2_]	*F*(000) = 1576
*M**_r_* = 383.44	*D*_x_ = 1.540 Mg m^−^^3^
Monoclinic, *C*2/*c*	Mo *K*α radiation, λ = 0.71073 Å
*a* = 16.8070 (8) Å	Cell parameters from 3218 reflections
*b* = 14.4212 (6) Å	θ = 2.0–26.0°
*c* = 15.5669 (9) Å	µ = 1.54 mm^−^^1^
β = 118.744 (4)°	*T* = 200 K
*V* = 3308.1 (3) Å^3^	Block, blue
*Z* = 8	0.10 × 0.08 × 0.07 mm

### Data collection

**Table:**

Stoe IPDS-2 diffractometer	θ_max_ = 26.0°, θ_min_ = 2.0°
ω scans	*h* = −20→19
3218 measured reflections	*k* = −17→17
3218 independent reflections	*l* = −2→19
2767 reflections with *I* > 2σ(*I*)	

### Refinement

**Table:**

Refinement on *F*^2^	0 restraints
Least-squares matrix: full	Hydrogen site location: mixed
*R*[*F*^2^ > 2σ(*F*^2^)] = 0.039	H-atom parameters constrained
*wR*(*F*^2^) = 0.105	*w* = 1/[σ^2^(*F*_o_^2^) + (0.0632*P*)^2^ + 1.3645*P*] where *P* = (*F*_o_^2^ + 2*F*_c_^2^)/3
*S* = 1.07	(Δ/σ)_max_ = 0.001
3218 reflections	Δρ_max_ = 0.72 e Å^−^^3^
178 parameters	Δρ_min_ = −0.40 e Å^−^^3^

### Special details

**Table:**

Geometry. All esds (except the esd in the dihedral angle between two l.s. planes) are estimated using the full covariance matrix. The cell esds are taken into account individually in the estimation of esds in distances, angles and torsion angles; correlations between esds in cell parameters are only used when they are defined by crystal symmetry. An approximate (isotropic) treatment of cell esds is used for estimating esds involving l.s. planes.
Refinement. Refined as a 2-component twin.

### Fractional atomic coordinates and isotropic or equivalent isotropic displacement parameters (Å^2^)

**Table:**

	*x*	*y*	*z*	*U*_iso_*/*U*_eq_	
Co1	0.500000	0.35611 (3)	0.750000	0.04070 (15)	
Co2	0.000000	0.64084 (3)	0.250000	0.04322 (15)	
N1	0.38555 (14)	0.28706 (15)	0.69242 (15)	0.0454 (5)	
C1	0.30981 (16)	0.26507 (16)	0.65171 (17)	0.0399 (5)	
S1	0.20412 (4)	0.23531 (6)	0.59318 (5)	0.0602 (2)	
N2	0.11433 (14)	0.70960 (15)	0.30776 (16)	0.0488 (5)	
C2	0.19017 (16)	0.73153 (16)	0.34319 (17)	0.0414 (5)	
S2	0.29563 (4)	0.76089 (6)	0.39174 (6)	0.0609 (2)	
S11	0.52044 (4)	0.43897 (5)	0.88607 (5)	0.05212 (19)	
C11	0.41958 (16)	0.49900 (16)	0.84464 (17)	0.0404 (5)	
N11	0.41916 (14)	0.58993 (14)	0.85125 (16)	0.0464 (5)	
H11	0.366818	0.618316	0.831086	0.056*	
C12	0.5004 (2)	0.64714 (19)	0.8938 (3)	0.0603 (7)	
H12A	0.539073	0.629383	0.962335	0.090*	
H12B	0.483201	0.712509	0.890419	0.090*	
H12C	0.533658	0.638091	0.857165	0.090*	
N12	0.34179 (14)	0.45328 (15)	0.80762 (17)	0.0525 (5)	
H12	0.346693	0.392461	0.809838	0.063*	
C13	0.25300 (16)	0.4945 (2)	0.7730 (2)	0.0596 (7)	
H13A	0.247660	0.518724	0.828810	0.089*	
H13B	0.206126	0.447472	0.738940	0.089*	
H13C	0.245126	0.545309	0.727713	0.089*	
S21	−0.01638 (5)	0.55786 (6)	0.36690 (6)	0.0628 (2)	
C21	0.08297 (16)	0.49509 (18)	0.42795 (17)	0.0448 (5)	
N21	0.08038 (14)	0.40531 (15)	0.43729 (16)	0.0494 (5)	
H21	0.131208	0.377325	0.478147	0.059*	
C22	−0.0021 (2)	0.3505 (2)	0.3986 (3)	0.0710 (9)	
H22A	−0.037573	0.369132	0.430511	0.107*	
H22B	0.013644	0.284667	0.411331	0.107*	
H22C	−0.037934	0.360733	0.327816	0.107*	
N22	0.16179 (14)	0.53884 (16)	0.46741 (18)	0.0562 (6)	
H22	0.158916	0.599750	0.468078	0.067*	
C23	0.24956 (16)	0.4953 (2)	0.5241 (2)	0.0584 (7)	
H23A	0.254623	0.471054	0.585312	0.088*	
H23B	0.297563	0.541035	0.538953	0.088*	
H23C	0.255899	0.444206	0.486232	0.088*	

### Atomic displacement parameters (Å^2^)

**Table:**

	*U*^11^	*U*^22^	*U*^33^	*U*^12^	*U*^13^	*U*^23^
Co1	0.0279 (2)	0.0390 (3)	0.0494 (3)	0.000	0.0138 (2)	0.000
Co2	0.0258 (2)	0.0420 (3)	0.0532 (3)	0.000	0.0121 (2)	0.000
N1	0.0382 (11)	0.0428 (11)	0.0500 (11)	−0.0038 (9)	0.0171 (9)	0.0013 (9)
C1	0.0369 (12)	0.0355 (11)	0.0441 (11)	−0.0007 (9)	0.0169 (10)	0.0046 (9)
S1	0.0349 (4)	0.0658 (5)	0.0642 (4)	−0.0127 (3)	0.0112 (3)	0.0176 (3)
N2	0.0359 (11)	0.0480 (12)	0.0564 (12)	−0.0041 (9)	0.0173 (9)	−0.0074 (9)
C2	0.0343 (12)	0.0383 (12)	0.0472 (12)	−0.0009 (9)	0.0162 (10)	−0.0027 (10)
S2	0.0338 (4)	0.0657 (5)	0.0756 (5)	−0.0133 (3)	0.0202 (3)	−0.0155 (4)
S11	0.0319 (3)	0.0517 (4)	0.0584 (4)	0.0053 (3)	0.0103 (3)	−0.0126 (3)
C11	0.0342 (12)	0.0430 (13)	0.0427 (11)	−0.0007 (9)	0.0174 (10)	−0.0042 (9)
N11	0.0359 (10)	0.0408 (11)	0.0594 (12)	0.0012 (8)	0.0204 (9)	0.0004 (9)
C12	0.0480 (16)	0.0447 (15)	0.0811 (19)	−0.0081 (12)	0.0252 (14)	0.0004 (13)
N12	0.0337 (11)	0.0430 (11)	0.0722 (14)	−0.0023 (9)	0.0185 (10)	−0.0081 (10)
C13	0.0315 (15)	0.065 (2)	0.0759 (18)	0.0008 (11)	0.0205 (13)	−0.0055 (14)
S21	0.0330 (3)	0.0697 (5)	0.0821 (5)	0.0105 (3)	0.0248 (3)	0.0292 (4)
C21	0.0336 (12)	0.0530 (15)	0.0435 (12)	−0.0003 (10)	0.0152 (10)	0.0039 (10)
N21	0.0381 (11)	0.0438 (12)	0.0560 (12)	−0.0009 (9)	0.0145 (10)	−0.0041 (9)
C22	0.0503 (18)	0.0550 (18)	0.094 (2)	−0.0149 (14)	0.0233 (16)	−0.0172 (16)
N22	0.0337 (11)	0.0455 (12)	0.0771 (15)	0.0016 (9)	0.0169 (11)	0.0117 (11)
C23	0.0309 (15)	0.061 (2)	0.0665 (16)	0.0046 (11)	0.0103 (12)	0.0036 (13)

### Geometric parameters (Å, º)

**Table:**

Co1—N1^i^	1.959 (2)	C2—S2	1.614 (2)
Co1—N1	1.959 (2)	S11—C11	1.728 (2)
Co1—S11	2.3093 (7)	C11—N11	1.316 (3)
Co1—S11^i^	2.3093 (7)	C11—N12	1.323 (3)
Co2—N2^ii^	1.955 (2)	N11—C12	1.454 (4)
Co2—N2	1.955 (2)	N12—C13	1.448 (3)
Co2—S21	2.3024 (8)	S21—C21	1.728 (3)
Co2—S21^ii^	2.3024 (8)	C21—N21	1.306 (3)
N1—C1	1.161 (3)	C21—N22	1.322 (3)
C1—S1	1.616 (2)	N21—C22	1.451 (4)
N2—C2	1.163 (3)	N22—C23	1.448 (3)
			
N1^i^—Co1—N1	118.89 (13)	C2—N2—Co2	165.2 (2)
N1^i^—Co1—S11	99.39 (6)	N2—C2—S2	179.3 (2)
N1—Co1—S11	111.28 (6)	C11—S11—Co1	103.32 (8)
N1^i^—Co1—S11^i^	111.28 (6)	N11—C11—N12	119.3 (2)
N1—Co1—S11^i^	99.39 (6)	N11—C11—S11	120.75 (18)
S11—Co1—S11^i^	117.68 (4)	N12—C11—S11	119.95 (18)
N2^ii^—Co2—N2	119.05 (13)	C11—N11—C12	124.2 (2)
N2^ii^—Co2—S21	99.35 (7)	C11—N12—C13	125.6 (2)
N2—Co2—S21	111.39 (7)	C21—S21—Co2	104.82 (8)
N2^ii^—Co2—S21^ii^	111.39 (7)	N21—C21—N22	120.1 (2)
N2—Co2—S21^ii^	99.35 (7)	N21—C21—S21	120.37 (18)
S21—Co2—S21^ii^	117.37 (5)	N22—C21—S21	119.5 (2)
C1—N1—Co1	165.2 (2)	C21—N21—C22	124.8 (2)
N1—C1—S1	178.9 (2)	C21—N22—C23	125.1 (2)

Symmetry codes: (i) −*x*+1, *y*, −*z*+3/2; (ii) −*x*, *y*, −*z*+1/2.

### Hydrogen-bond geometry (Å, º)

**Table:**

*D*—H···*A*	*D*—H	H···*A*	*D*···*A*	*D*—H···*A*
N11—H11···S1^iii^	0.88	2.65	3.341 (2)	136
N12—H12···N1	0.88	2.69	3.282 (3)	126
N12—H12···S2^iv^	0.88	2.88	3.582 (2)	138
C13—H13*B*···S21^v^	0.98	2.80	3.578 (3)	137
N21—H21···S1	0.88	2.61	3.372 (2)	146
C22—H22*A*···S2^vi^	0.98	3.01	3.591 (3)	120
C22—H22*A*···S21^v^	0.98	3.02	3.752 (4)	132
N22—H22···S2^vii^	0.88	2.79	3.486 (3)	137

Symmetry codes: (iii) −*x*+1/2, *y*+1/2, −*z*+3/2; (iv) *x*, −*y*+1, *z*+1/2; (v) −*x*, −*y*+1, −*z*+1; (vi) *x*−1/2, *y*−1/2, *z*; (vii) −*x*+1/2, −*y*+3/2, −*z*+1.
