# Supplementary figures and images for: Crystal structure of bis­(N,N′-di­methyl­thio­urea-κS)bis­(thio­cyanato-κN)cobalt(II)
Source: Acta Crystallogr E Crystallogr Commun. 2020 Aug 18;76(Pt 9):1476–81. doi: 10.1107/S2056989020011111 (PMC7472772; doi:10.1107/S2056989020011111)

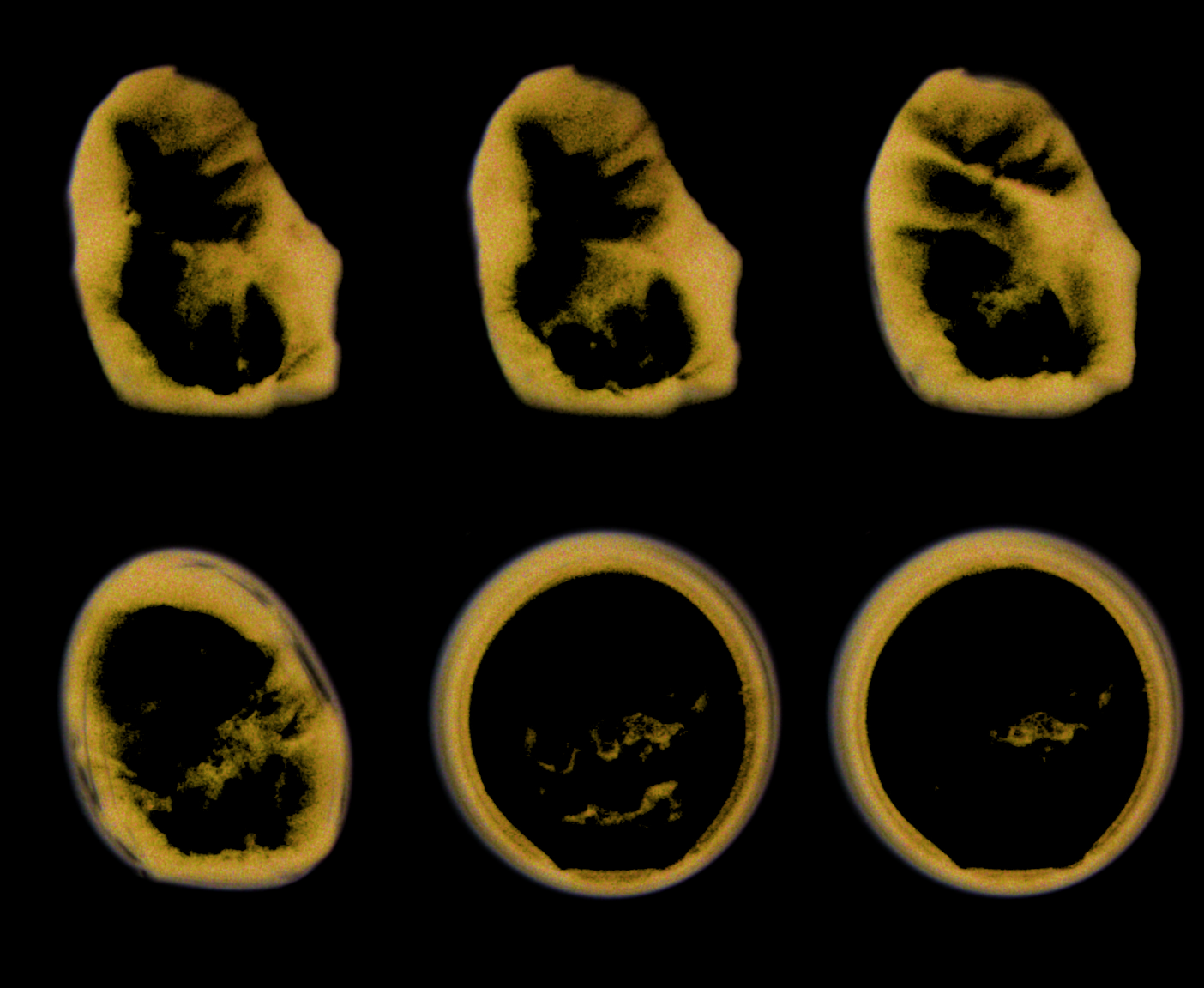

Supplement: Supplementary file 8 [file e-76-01476-sup8.tif]
